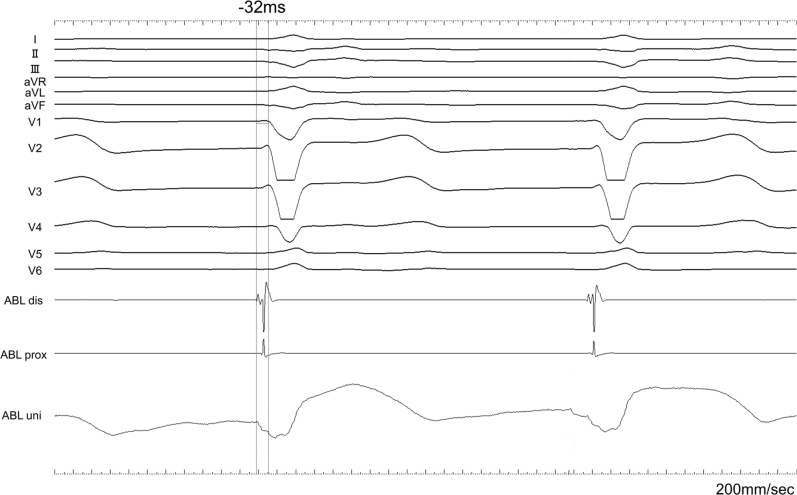# Ablation of right bundle branch Purkinje-origin ventricular parasystole: The role of omnipolar frequency mapping

**DOI:** 10.1016/j.hrcr.2025.07.027

**Published:** 2025-08-06

**Authors:** Yoshihiro Sobue, Taishi Fukushima, Eiichi Watanabe, Hideo Izawa

**Affiliations:** 1 Department of Cardiology, Fujita Health University Bantane Hospital, Nagoya, Aichi, Japan; 2 Department of Cardiology, Fujita Health University School of Medicine, Toyoake, Aichi, Japan

**Keywords:** Ventricular parasystole, His-Purkinje system, Omnipolar Technology Near Field, High-resolution mapping, Right bundle branch arrhythmia

**Key Teaching Points**

• Parasystoles originating from the His-Purkinje system can be successfully ablated by targeting Purkinje-like potentials using high-resolution mapping techniques.
• Omnipolar Technology Near Field frequency mapping (>400 Hz) provides real-time identification of high-frequency potentials at the ectopic site, enhancing procedural accuracy.
• Fusion beats and entrance block are hallmark electrocardiographic findings of ventricular parasystoles, aiding diagnosis and procedural planning.
• A combined approach using activation mapping, pace mapping, and spectral frequency analysis maximizes effectiveness for the ablation of Purkinje-mediated arrhythmias.

## Introduction

Ventricular parasystoles are a rare cardiac arrhythmia characterized by an ectopic pacemaker that discharges independently because of entrance block.^1^ On the surface electrocardiogram (ECG), ventricular parasystole is characterized by premature ventricular complexes (PVCs) with variable coupling intervals to preceding QRS complexes, the presence of at least 3 consecutive PVCs occurring at a constant intrinsic interval (“marching out”), and occasional fusion beats. These features reflect the existence of an ectopic pacemaker protected by entrance block, allowing it to discharge independently of the surrounding myocardium.^1^ Although generally considered benign, ventricular parasystoles can, in some cases, contribute to a significant arrhythmic burden. The His-Purkinje system plays a crucial role in coordinating ventricular depolarization, yet its involvement in ventricular parasystoles remains incompletely understood.^2^

Advances in 3-dimensional electroanatomic mapping techniques have significantly improved our ability to identify and target ectopic foci responsible for ventricular arrhythmias.^3^ High-density mapping has emerged as a promising approach for refining the localization of Purkinje-mediated arrhythmias. The frequency mapping method used in the Omnipolar Technology Near Field (OTNF) analysis involves real-time detection and visualization of high-frequency potentials, allowing more precise differentiation of arrhythmic foci. Recent studies have demonstrated the efficacy of frequency mapping in identifying Purkinje-related triggers in ventricular fibrillation and complex arrhythmias, thereby enhancing ablation success rates.^4^ In this report, we describe a unique case of ventricular parasystole originating from the Purkinje fibers originating from the right bundle branch. By using the OTNF frequency analysis, we were able to accurately identify the ectopic source and successfully eliminate the arrhythmia through targeted catheter ablation. This case highlighted the clinical utility of a frequency analysis in mapping ventricular parasystoles and reinforced its role as a valuable adjunct in the treatment of His-Purkinje–mediated arrhythmias.

## Case report

**Figure 1:**
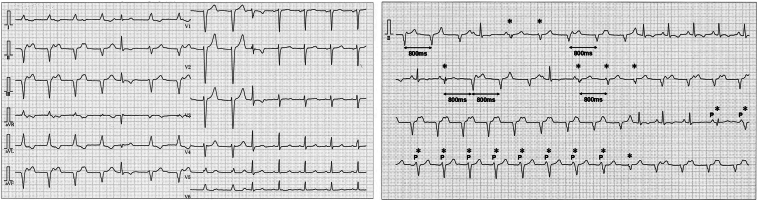
Surface electrocardiogram demonstrating ventricular parasystole with intermittent fusion beats. The ectopic QRS complexes exhibit complete left bundle branch block morphology with a superior axis. Fusion beats are denoted by *asterisks*. Sinus P waves are labeled as “P” to indicate an underlying independent sinus rhythm. The parasystolic cycle length was constant at 800 ms, confirming a dissociated ectopic pacemaker activity.

A 27-year-old woman was referred for the evaluation of frequent PVCs. She remained asymptomatic but exhibited persistent ventricular ectopy on routine electrocardiography. The 12-lead ECG revealed ventricular arrhythmia occurring independently of sinus rhythm, with intermittent fusion beats (Figure 1). The ectopic QRS morphology was consistent with a complete left bundle branch block pattern and superior axis, suggesting an origin from the right bundle branch system. The cycle length of the parasystolic beats was recorded at 800 ms, confirming its distinct rhythm independent of sinus node influence. Based on surface ECG findings, the diagnosis of ventricular parasystole was made. The parasystolic beats demonstrated a constant morphology distinct from sinus beats, with variable coupling intervals to the preceding QRS complexes. Furthermore, multiple beats were observed “marching out” at fixed intervals, consistent with the diagnostic criteria for parasystole.^1^ Occasional fusion beats were also observed, supporting the presence of an independent ectopic focus protected by entrance block.

**Figure 2:**
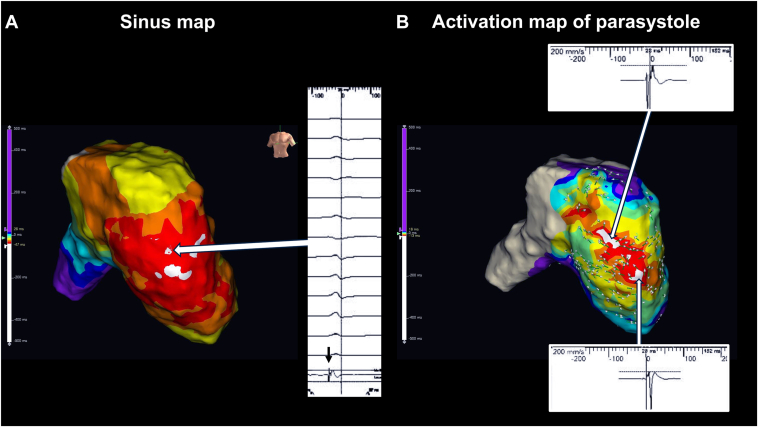
**A:** Sinus rhythm activation map demonstrating the earliest activation site in the right ventricular free wall (*white arrow*). High-frequency potentials suggestive of Purkinje-like potentials were recorded at this site. **B:** The activation map of ventricular parasystole identified 2 earliest activation sites located in the right ventricular free wall: one at the basal site and another at the apical site. Both sites exhibited potentials preceding the onset of the parasystolic QRS complex by 32 ms; however, the basal site showed a sharp, high-frequency Purkinje-like potential whereas the apical site exhibited a dull, low-frequency potential.

**Figure 3:**
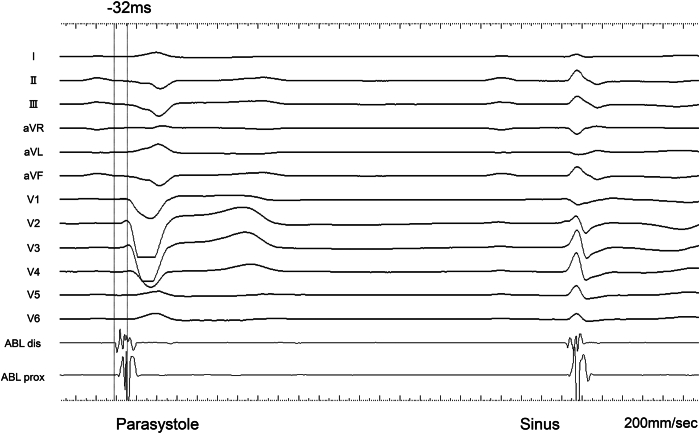
Intracardiac electrogram (EGM) recording demonstrating a high-frequency potential preceding the QRS complex by 32 ms recorded at a sweep speed of 200 mm/s. The EGM was obtained from the basal site identified as the high-frequency target on the frequency map during ablation catheter placement.

Electrophysiologic mapping was conducted using the EnSite X (Abbott) system with an Advisor HD Grid catheter. The activation map and vector analysis identified 2 earliest activation sites of ventricular parasystole, located at the basal and apical segments of the free wall (Figure 2). Both sites exhibited discrete prepotentials that consistently preceded the onset of the parasystolic QRS complex by 32 ms (Figure 3 and Supplemental Figure 1). After acquisition of the activation map, pace mapping was performed to evaluate the morphological similarity between the spontaneous parasystolic beats and the paced QRS complexes at the site of earliest activation. The matching score was calculated using a morphology-matching algorithm that compared 12-lead ECG waveforms. The *analysis window* was defined from the onset to the end of the QRS complex, explicitly excluding the pacing artifact to avoid waveform distortion. Time alignment between the paced and spontaneous beats was automatically optimized on the basis of the point of maximal slope (dV/dt) to ensure accurate superimposition.

**Figure 4:**
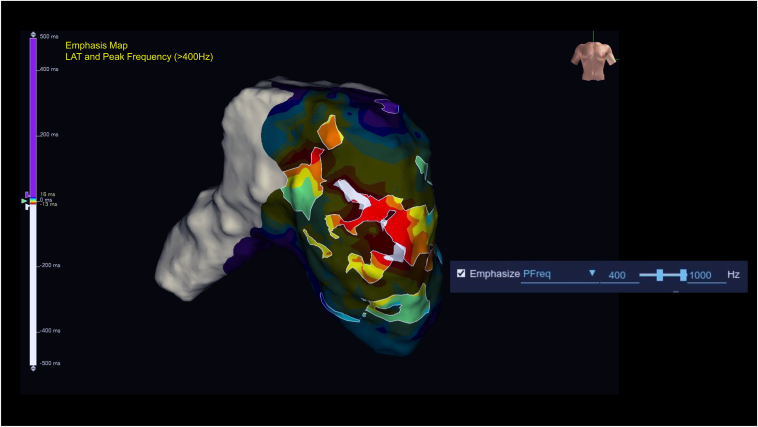
Combined activation and frequency map (threshold > 400 Hz). An activation map overlaid with a frequency map, allowing simultaneous visualization of early activation timing and high-frequency components. Activation mapping identified 2 earliest activation sites at the basal and apical right ventricular free wall. The addition of frequency mapping highlighted only the basal site exhibited high-frequency potentials. LAT = local activation time.

**Figure 5:**
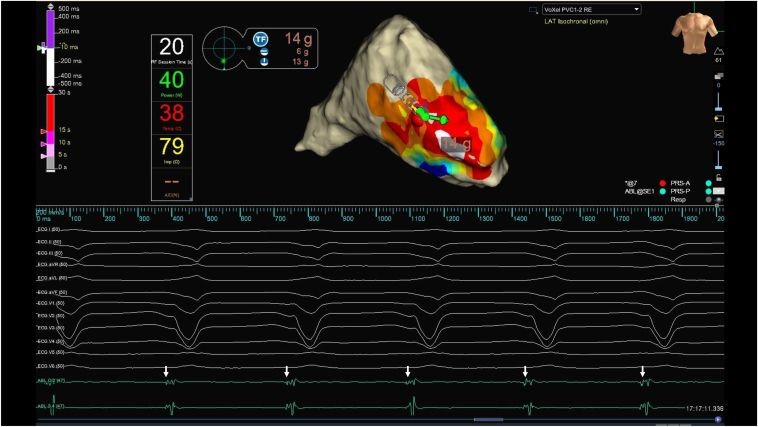
Radiofrequency ablation targeting the earliest activation site identified by activation mapping and an Omnipolar Technology Near Field (OTNF) analysis. The upper scale bar indicates activation prematurity relative to the QRS onset, whereas the lower scale bar represents ablation duration.

**Figure 6:**
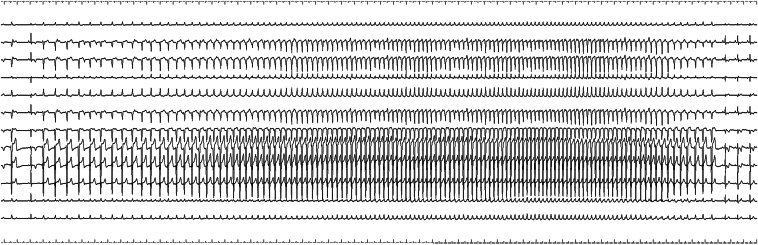
Electrocardiographic recordings during radiofrequency ablation. Immediately after the energy application, acceleration of the parasystolic rhythm was observed, as indicated by the shortening of the cycle length. Subsequently, the cycle length gradually prolonged, leading to the complete termination of the parasystole.

**Supplementary Figure 2:**
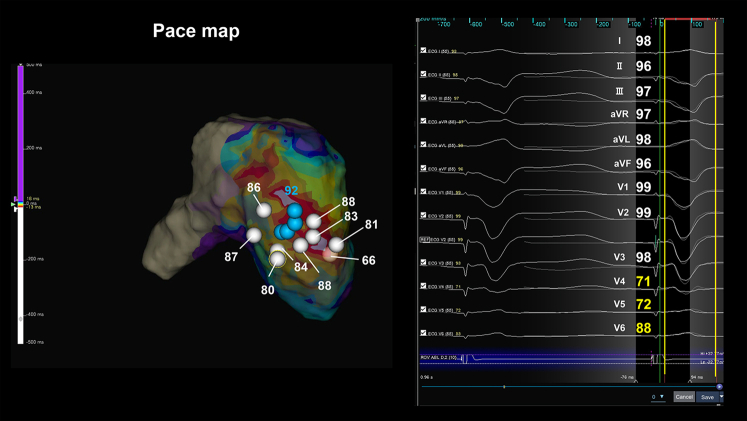


**Supplementary Figure 3:**
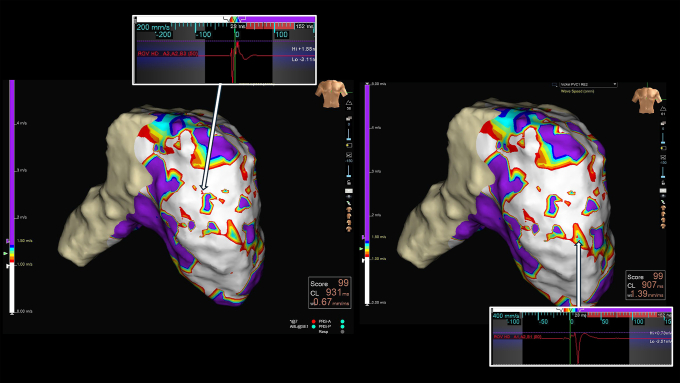


At these earliest activation sites, pace mapping achieved a high but suboptimal match with the clinical parasystolic beat, yielding a maximum score of 92 of 100, suggesting a close but not exact reproduction of the ectopic focus (Supplemental Figure 2). In addition to activation and pace mapping, the arrhythmic substrate was systematically characterized using activation vector mapping, conduction velocity mapping, and high-frequency component mapping with omnipolar technology to improve the precision of localizing the arrhythmic focus. The frequency mapping algorithm automatically detects the peak frequency within a user-defined window of interest by applying wavelet-based decomposition to the entire electrogram.^4^ Figure 4 shows an activation map overlaid with a frequency map, allowing simultaneous visualization of early activation timing and high-frequency components. Using the frequency map with a threshold set to >400 Hz, only the basal ectopic site was prominently highlighted as a discrete high-frequency zone, suggesting the presence of Purkinje-like potentials, whereas the apical earliest activation site exhibited no significant high-frequency potentials (Figure 4). In contrast, the conduction velocity at the basal and apical earliest activation sites was measured at 0.67 and 1.39 m/s, respectively, both falling within the physiological range and thus not indicating any localized conduction slowing (Supplemental Figure 3).^5^ Radiofrequency ablation was performed at the basal ectopic site using a TactiFlex catheter (Abbott) with an initial power setting of 40 W (Figure 5). On applying an energy application, an immediate acceleration of the parasystolic rhythm was observed (Figure 6). As ablation continued, the cycle length of the parasystolic beats gradually prolonged, culminating in complete suppression of the arrhythmia. The patient remained free of recurrent parasystolic activity throughout the postprocedure monitoring period.

## Discussion

This case highlighted a rare and significant finding: successful ablation of ventricular parasystoles originating from Purkinje-like potentials in the free wall of the right ventricle. Through precise mapping and ablation, the parasystole exhibited an acceleration before terminating and was followed by its complete elimination. That response strongly suggested that the targeted Purkinje-like potentials were the source of the arrhythmia, providing critical insight into the mechanisms of ventricular parasystoles and their management.

Parasystoles are driven by abnormal automaticity, which arises from a persistently depolarized membrane potential during phase 4. This sustained depolarization enhances the calcium channel activity, facilitating spontaneous depolarization and ectopic pacemaker activity.^6^ As a result, the parasystolic focus can discharge independently of the surrounding myocardium, contributing to its characteristic entrance block and resistance to overdrive suppression. Additionally, entrance block prevents overdrive suppression by insulating the ectopic focus from surrounding myocardial impulses. This unique combination explains why parasystoles can persist despite the dominant rhythm. Although the parasystolic activity appeared to diminish during the last portion of the ECG recording, this likely reflects intermittent fusion or modulation, rather than suppression, and is consistent with the behavior of modulated parasystole with partial entrance block.^1^^,^^7^^,^^8^ The observed acceleration during ablation in this case supported the concept of localized abnormal automaticity within the Purkinje system. This phenomenon, which is often referred to as “entrainment-like acceleration,” has been described in other arrhythmias involving automatic foci but is rarely documented in ventricular parasystoles.^2^ These findings reinforce the critical role of Purkinje fibers in arrhythmogenesis, particularly in the context of ventricular parasystoles originating from anatomically challenging regions.

Although ventricular parasystoles arising from the His-Purkinje system have been documented, reports specifically identifying their origin from the distal right bundle branch remain limited.^9^ Previous studies have demonstrated the value of advanced mapping techniques for left fascicular parasystoles, emphasizing the importance of identifying early potentials during arrhythmia mapping.^6^ Another report described parasystoles as a manifestation of autonomous ectopic pacemakers protected by entrance block, with modulation further complicating their recognition. These insights underline the interplay between entrance block and automaticity as central to the pathogenesis of parasystoles.

The application of OTNF frequency mapping in this case played a crucial role in precisely localizing the ectopic Purkinje focus. Frequency mapping, as an emerging technology, has been shown to improve the accuracy of arrhythmic substrate identification, particularly in complex Purkinje-mediated arrhythmias. By enabling real-time detection of high-frequency potentials, this method facilitates a more targeted ablation strategy and reduces the procedural duration. Recent studies have reported that OTNF frequency mapping, which has been recently introduced into clinical practice, provides superior spatial resolution and allows for a more refined differentiation of viable Purkinje tissue from adjacent myocardial substrates.^4^ Similarly, in ventricular arrhythmias, automated peak frequency (PF) annotation has been reported to identify deceleration zones and potential ablation targets, typically using a frequency threshold above 250 Hz. However, an optimal peak frequency cutoff has not been universally standardized and likely varies depending on myocardial thickness, electrogram morphology, and anatomical location. In this case—mapping a focus on the thin right ventricular free wall—we empirically adjusted the threshold to 400 Hz to enhance the detection of sharp near-field signals and reduce interference from low-frequency far-field components. To our knowledge, this is the first description of PF-guided ablation in the right ventricular free wall. Further investigation is warranted to validate its clinical utility and to determine optimal frequency thresholds across different cardiac regions.^10^

In our case, the initial activation map demonstrated 2 discrete earliest activation sites: one located in the basal free wall and the other toward the apical region. However, PF analysis revealed focal high-frequency activity exceeding 400 Hz limited to the basal site, whereas no significant high-frequency potentials were recorded at the apical site. This differentiation, achievable only through frequency analysis, provided critical additional confirmation of the arrhythmogenic focus. These findings, in combination with activation timing, electrogram morphology, and a high but suboptimal pace mapping match (92%), supported the selection of the basal region as the most likely site of origin. This approach is especially beneficial in arrhythmias where conventional activation mapping may fail to adequately distinguish between ectopic foci and surrounding conduction tissue.

One major challenge in this case was the suboptimal pace mapping match at the earliest activation site. This can be attributed to intrinsic properties of parasystoles, including entrance block and phase 4 block. Entrance block limits impulse penetration into the ectopic focus, altering conduction during pacing. Furthermore, refractoriness of the adjacent myocardium impairs exact reproduction of the clinical QRS morphology. In our case, pace mapping yielded a high but incomplete match (92%), consistent with prior reports that pacing may capture both Purkinje fibers and adjacent myocardium, slightly modifying the QRS configuration. Additionally, absent activation of neighboring beats suggests that entrance block may have reduced pacing capture, explaining the observed discrepancy.

The integration of OTNF mapping with traditional activation mapping represented a significant advancement in the approach to catheter ablation of ventricular parasystoles. By providing detailed insight into the electrophysiological properties of arrhythmic foci, this combined strategy enhances the precision of ablation procedures, particularly in cases involving the His-Purkinje system. Future studies should further explore the application of OTNF frequency mapping in various arrhythmias to validate its efficacy and establish standardized protocols for its use in clinical practice.

The successful elimination of ventricular parasystoles in this case underscored the potential value of OTNF frequency mapping as an adjunct to conventional mapping techniques for Purkinje-related arrhythmias. Future research should focus on refining this technique, optimizing frequency mapping thresholds, and expanding its application to other conduction system arrhythmias.

## Conclusion

This case demonstrated the successful catheter ablation of ventricular parasystoles originating from Purkinje fibers of the distal right bundle branch. The use of OTNF frequency mapping facilitated a precise localization of the ectopic focus and guided effective ablation. Those findings highlighted the potential of targeted ablation in this region and the clinical utility of OTNF mapping in Purkinje-mediated arrhythmias.

## Disclosures

The authors have no conflicts of interest to disclose.

## Figures and Tables

**Supplementary Figure 1:**