# Supplementary material for: Ablation of right bundle branch Purkinje-origin ventricular parasystole: The role of omnipolar frequency mapping
Source: HeartRhythm Case Rep. 2025 Aug 6;11(10):1096–101. doi: 10.1016/j.hrcr.2025.07.027 (PMC12666964; doi:10.1016/j.hrcr.2025.07.027)
Supplement: Supplementary Figure Legends [file mmc1.docx]

**Supplemental Figure 1.**

Unipolar electrogram recorded at the basal site. The unipolar electrogram at the basal right ventricular free wall shows a pure QS pattern.

**Supplemental Figure 2. Pace mapping results at the earliest activation site.**
The maximum matching score was 92 out of 100, with lower matching scores observed in the surface ECG leads V4–V6.

**Supplemental Figure 3. Conduction velocity map.**
The basal and apical sites showed conduction velocities of 0.67 m/s and 1.39 m/s, respectively, both within the physiological range without localized slowing.
